# *Brucella abortus* BspJ Is a Nucleomodulin That Inhibits Macrophage Apoptosis and Promotes Intracellular Survival of *Brucella*

**DOI:** 10.3389/fmicb.2020.599205

**Published:** 2020-11-12

**Authors:** Zhongchen Ma, Ruirui Li, Ruirui Hu, Xiaoyu Deng, Yimei Xu, Wei Zheng, Jihai Yi, Yong Wang, Chuangfu Chen

**Affiliations:** ^1^International Joint Research Center for Animal Health Breeding, College of Animal Science and Technology, Shihezi University, Shihezi, China; ^2^Collaborative Innovation Center for Prevention and Control of High Incidence Zoonotic Infectious Diseases in Western China, College of Animal Science and Technology, Shihezi University, Shihezi, China; ^3^Key Laboratory of Control and Prevention of Animal Disease, Xinjiang Production & Construction Corps, College of Animal Science and Technology, Shihezi University, Shihezi, China; ^4^College of Life Science, Shihezi University, Shihezi, China; ^5^Xinjiang Center for Disease Control and Prevention, Urumqi, China

**Keywords:** *Brucella*, nucleomodulin, BspJ, yeast two-hybrid, NME2, CKB

## Abstract

To date, a variety of *Brucella* effector proteins have been found to mediate host cell secretion, autophagy, inflammation, and other signal pathways, but nuclear effector proteins have not yet been reported. We identified the first *Brucella* nucleomodulin, BspJ, and we screened out the BspJ interaction host proteins NME/NM23 nucleoside diphosphate kinase 2 (NME2) and creatine kinase B (CKB) through yeast two-hybrid and co-immunoprecipitation assays. These proteins are related to the host cell energy synthesis, metabolism, and apoptosis pathways. *Brucella* nucleomodulin BspJ will decrease the expression level of NME2 and CKB. In addition, BspJ gene deletion strains promoted the apoptosis of macrophages and reduced the intracellular survival of *Brucella* in host cells. In short, we found nucleomodulin BspJ may directly or indirectly regulate host cell apoptosis through the interaction with NME2 and CKB by mediating energy metabolism pathways in response to the intracellular circulation of *Brucella* infection, but the mechanism needs further study.

## Introduction

*Brucella* infects animals and human beings and causes Brucellosis (Atluri et al., 2011), a major zoonotic disease endemic in more than 170 countries and regions of the world (Avilacalderon et al., 2013). Brucellosis is classified as an important reemerging disease of humans and animals in China (Zhou et al., 2020), where it has shown an upward trend in recent years (Zheng et al., 2018). At present, Brucellosis has been reported in many mammals (Moreno, 2014); except for some antibiotics and corticosteroids, there is no effective specificity treatment for the human brucellosis.

Animals and humans infected with *Brucella* have the same pathological and physiological characteristics at the cellular level. *Brucella*’s survival, proliferation and parasitism depend on its intracellular circulation (including in macrophages and dendritic cells) in the host (Atluri et al., 2011; Celli, 2015). During *Brucella* infection of the host, after being engulfed by phagocytes or invading non-phagocytic cells, *Brucella* firstly hides in a membrane-bound compartment (*Brucella*-containing vacuole, BCV) (Comerci et al., 2001; Celli et al., 2003), completing the maturation process similar to that of the complete phagosome. Due to its endosome properties in last process, the bacterial structure is known as an endosomal BCV (eBCV) (Boschiroli et al., 2002; Celli et al., 2003; Starr et al., 2008). Subsequently, with the inversion of the eBCV membrane and the accumulation of endoplasmic reticulum (ER)-derived membranes, the eBCV is transformed into ER-derived organelles called replicative BCV (rBCV) (Starr et al., 2012). Finally, with *Brucella* proliferates in large numbers in rBCVs, it forms a recombinant rBCV called an autophagy BCV (aBCV) that releases mature *Brucella* to complete its intracellular circulation process and infect new cells (Celli, 2019).

The *Brucella* VirB type IV secretion system (T4SS) was obtained by homologous identification with the plant pathogen *Agrobacterium*, and its expression is an important indicator of *Brucella*’s virulence (O’Callaghan et al., 1999; Sieira et al., 2000; Delrue et al., 2001). Numerous studies have shown that T4SS plays an important role throughout the *Brucella* cycle. There are 15 kinds of VirB T4SS effectors that have been identified, and their functions and effects can be used to interpret some mechanisms of *Brucella* proliferation and intracellular survival (Celli, 2019). VceC is expressed and secreted by VirB T4SS, which can bind to the ER chaperone Grp78/BiP and induce the unfolded protein response (UPR), triggering an inflammatory response (de Jong et al., 2013). TcpB/BtpA/Btp1 is a *Brucella* VirB T4SS secreted effector protein containing the TIR [Toll/interleukin-1 (IL-1) receptor] domain that can down-regulate the expression of pro-inflammatory factors (Alaidarous et al., 2014; Jakka et al., 2017) and inhibit inflammation. Once *Brucella* forms rBCVs, it will also induce UPR and promote bacterial proliferation (Smith et al., 2013). The T4SS effector proteins BspA, BspB, and BspF can inhibit the secretion of host proteins and promote the proliferation of *Brucella* (Myeni et al., 2013; Miller et al., 2017). BspB can also interact with the conserved oligomeric Golgi (COG) complex (Miller et al., 2017). The T4SS effector RicA can control the production process of rBCV, which can bind to the GDP binding domain of Rab2 (de Barsy et al., 2011), but its mode of action is unclear.

Nucleomodulins are a family of effectors produced by bacterial pathogens to control host transcription or other nuclear processes. However, nucleomodulin of *Brucella* has not been reported (Lebreton et al., 2014; Farris et al., 2016, 2017). Secretory protein BspJ (BAB2_0119, updated ID BAB_RS26920) of *Brucella* is a putative effector protein that may enter the host cell nucleus (Myeni et al., 2013). Some intracellular bacteria can secrete and deliver effector proteins or nucleoprotein complexes to exert virulence. Similar to those proteins that cause bacterial virulence (Juhas et al., 2008; Green and Mecsas, 2016), we speculate that *Brucella* can also transport effector proteins that function in the host cell nucleus. We identified the expression of BspJ in the nucleus of the host cell consistent with reported earlier by Myeni et al. (2013). Using the HEK293T cell genome as a library, we discovered four proteins that interact with BspJ (MIF, NME2, CKB, and RPL13) by means of yeast two-hybrid assays. We verified through co-immunoprecipitation (CO-IP) experiments that BspJ does interact with NME2 and CKB to regulate the energy metabolism pathways of host cells. BspJ reduced the expression levels of NME2 and CKB in HEK293T cells, and the knockdown of BspJ accelerated the apoptosis of macrophages and reduce the intracellular viability of *Brucella*. The results provide the foundation for subsequent functional studies of *Brucella* BspJ protein.

## Materials and Methods

### Strains, Cells, and Reagents

*Brucella abortus* strain 2308 was provided by the China Center for Disease Control and Prevention (Beijing, China), cultured in tryptone soya agar (TSA) or tryptone soya broth (TSB) (Oxoid, United Kingdom). *Brucella abortus* strain 2308 *BspJ* gene deletion strain (*Brucella abortus* ΔBspJ) and complementary strain (*Brucella abortus* pBspJ) were provided by Xinjiang Center for Disease Control and Prevention, where carried out all biosafety trials involved in this study. *Escherichia coli* DH5α standard strain was cultured in LB (Luria-Bertani) medium. HEK293T cells and macrophages RAW264.7 were provided by the Cell Resource Center, the Institute of Basic Medical Sciences of the Chinese Academy of Medical Sciences/Peking Union Medical College (Beijing, China), and were cultured in DMEM medium containing 10% fetal bovine serum (FBS, Gibco) (Thermo Fisher Scientific, United States) in a 5% CO_2_ incubator. Yeast AH109 (*MAT*α) (Clontech, United States) was cultured on SD plates (Coolaber, China).

### Amplification of *BspJ* Gene and Vector Construction

We searched the *BspJ* gene sequence in the Genbank database. Primer 5.0 was employed to design the primers used to amplify the target fragment (BspJ-F 5′-ATGAAGAGCTTGCAGTTTTC-3′, BspJ-R 5′-TTATCGATATGCCCGAGGTAC-3′). *Brucella abortus* was used to as a genome template for BspJ gene cloning. The C-terminal fusion His tag of the *BspJ* gene constructed to pDsRed2-C1 (Clontech, United States) and pcDNA3.1 (Invitrogen, United States) vectors. Using an endotoxin-free plasmid large-scale extraction kit (TIANGEN, China) to extract the plasmids pDsRed2-C1-BspJ and pcDNA3.1-BspJ. Meanwhile, the *BspJ* gene was constructed into the vector pGBKT7 (Clontech, United States).

### Location of BspJ Protein in HEK293T

The HEK293T cells were cultured on cell slides in a 12-well plate. When the cell density reached 80%, Lipofectamine 3000 (Invitrogen, United States) was used to transfer the constructed vector pDsRed2-C1-BspJ to HEK293T cells. Post-transfected 24 h, discarded the cell culture medium, and stained the nucleus with DAPI (Solarbio, China) for 5 min. This step was protected from light. Anti-fluorescence quencher (Solarbio, China) was then added. The location of the BspJ protein in HEK293T cells was observed under a confocal microscope (Nikon C2i+, Japan).

### Expression of BspJ Protein in the Host Cell

HEK293T cells were cultured in a cell culture plate at an order of magnitude of 1 × 10^7^ cells/well. When the cell density reached 80∼90%, Lipofectamine 3000 (Invitrogen, United States) was used to transfect the plasmid (pcDNA3.1-BspJ) into the HEK293T cell. After 48 h, a nuclear protein extraction kit (BestBio, China) was employed to extract the nuclear and cytoplasmic proteins. This was followed by SDS-PAGE (CWBIO, China) for western blot analysis using rabbit anti-6 × His tag antibody (Abcam, United States) (1:2000) as the primary antibody. Goat anti rabbit IgG H&L (Abcam, United States) (1:4000) was used as the secondary antibody, and a SuperSignal^TM^ West Femto Trial kit (Thermo Fisher Scientific, United States) was used for color developing. Nucleoprotein histone was used as control. The primary antibody Mouse Anti-Histone H3 Monoclonal Antibody (SinoBiological, China) (1:2000) and the secondary antibody Goat Anti-Mouse IgG H&L (Abcam, United States) (1: 4000) were used in the experiment.

### Construction of HEK293T cDNA Library

An Ultrapure RNA Kit (CWBIO, China) was used to extract total RNA from HEK293T cells following the manufacturer’s instructions. Fasttrack MAG beads (Thermo Fisher Scientific, United States) were used to isolate mRNA. Biotin-DSN-attB2/attB1 primers were added to synthesize a cDNA library. BP Clonase II Mixp (Thermo Fisher Scientific, United States) was added to recombine pDONR222 (ZYbscience, China) with cDNA, and then electro-transformed into *E. coli* DH10B (Novagen, United States). A PureLink^®^ HiPure Plasmid Filter Midiprep Kit (Thermo Fisher Scientific, United States) was employed to extract library plasmids. The library plasmids and pGADT7-DEST (Clontech, United States) were co-transformed to DH10B. Next, the bacterial stock solution was diluted 1000 times to identify the capacity of the cDNA library. The solution was spread on an LB plate (containing the corresponding antibiotic) and incubated overnight at 37°C. CFU/mL = number of clones on the plate/coating volume (μL) × 1000 × 1 × 10^3^ μL. Total CFU = CFU/mL × total volume of library bacteria (mL). The expected total size of the amplitude reaching 1.0 × 10^6^ CFU/mL is considered optimum. Twenty-four clones were randomly selected for colony PCR to identify the recombination rate and insert length. Primer sequences were as follows: T7, 5′-TAATACGACTCACTATAGGGCGAGCGCCG CCATG-3′ ADR, 5′-GTGAACTTGCGGGGTTTTTCAGTAT CTACGATT-3′. The detailed PCR parameters are shown in Supplementary Figure 1A.

### Detection of the Constructed HEK293T cDNA Library

After identification and calculation, the capacity of the HEK293T cDNA library was determined to be 5.02 × 10^6^ CFU/mL, and the total number of clones was 1.0 × 10^7^ CFU/mL, which reached the standards for yeast two-hybrid library construction and use. We identified the recombination rate and insert length of 24 random monoclonal colonies, and the target fragment was successfully amplified (Supplementary Figure 1C). Therefore, we concluded that we successfully constructed the HEK293T cDNA library.

### Yeast Two-Hybrid Screening of BspJ Interacting Proteins

The PEG/LiAc method was employed to transform pGBKT7-BspJ into yeast AH109 cells (MATα) (Clontech, United States), and then the self-activation and cytotoxicity of BspJ were verified. SD/–Leu/–Trp (DDO) plates (Coolaber, China) and SD/–Ade/–His/–Leu/–Trp (QDO) plates (Coolaber, China) were used to screen monoclonal colonies (>2 mm), and chosen colonies were placed in DDO liquid medium with shaking during the logarithmic growth phase (29°C, 200 rpm, 200 h) and then plated on the QDO medium. Both of the DDO plate and the QDO plate have aseptic spots indicated that the BspJ protein was toxic. DDO and QDO plates have plaques indicated that the BspJ protein had self-activation activity. DDO have white plaques and QDO have sterile plaques indicated that the BspJ protein had no self-activating activity.

The pGBKT7-BspJ vector was first transformed by the PEG/LiAc method, and the vector pGADT7-cDNA was co-transformed after screening and denaturation of salmon sperm DNA (Coolaber, China). Then select positive clones on DDO, QDO and SD/–Ade/–His/–Leu/–Trp/X-α-gal (QDO/X) (Coolaber, China) medium. The positive clones sequencing results were analyzed via BLAST (Basic local alignment search tool^1^).

### Switch Back Prey Plasmid and Confirmation of Positive Interactions in Yeast

The selected positive clones were extracted using a yeast plasmid extraction kit (Solarbio, China), and then transformed into *E. coli* DH5α (Collaborative Innovation Center for the Prevention and Control of Infectious Diseases in the Western Region, China). After culturing, the plasmid was extracted with a plasmid extraction kit (TIANGEN, China), and the plasmid and pGBKT7-BspJ were co-transformed into yeast AH109 cells (*MAT*α). The positive bacteria were screened by coating DDO, QDO, and QDO/X auxotrophic plates.

### Co-immunoprecipitation Test to Verify Protein-Protein Interaction

The selected protein gene C-terminal fusion Myc tag (-Myc) was constructed into the vector pcDNA3.1 (Invitrogen, United States), and the plasmid was extracted using an endotoxin-free plasmid large-scale extraction kit (TIANGEN, China). The pcDNA3.1-BspJ and -Myc plasmids were transfected into HEK293T cells with Lipofectamine 3000 (Invitrogen, United States). After 48 h, IP lysis buffer (Biosharp, China) (containing protein phosphatase inhibitor) was added to harvest the cell proteins. The protein solution was incubated with mouse Anti-6 × His tag antibody and mouse Anti-Myc tag antibody (Abcam, United States) (1:200) at low temperature (4°) for 3 h or overnight, and Protein G-Agarose (Roche, Germany) was added for 4° co-incubation after 3 h or overnight. The precipitate was collected by centrifugation, washed with PBS three times, mixed with SDS-PAGE protein loading solution (CWBIO, China), boiled for five minutes, and subjected to western blot analysis. Mouse Anti-6 × His Tag antibody/Mouse Anti-Myc tag antibody (1:1000) was used as the primary antibody; Goat Anti-Mouse IgG H&L (Abcam, United States) (1:2000) was used as the secondary antibody, and a SuperSignal^TM^ West Femto Trial Kit (Thermo Fisher Scientific, United States) was used for color developing.

### Bioinformatics Analysis of Protein Potential Functions

The protein transmembrane region and signal peptide prediction employed online software TMHMM Server v. 2.0^2^ and SignalP 4.1 Server^3^. Protein nuclear localization signal (NLS) analysis used online prediction software cNLS Mapper^4^. Protein nuclear output signal (NES) analysis used online software NetNES 1.1 Server^5^. The String software^6^, Gene Ontology (GO) database, and Kyoto Encyclopedia of Gene and Genomes (KEGG) database were used to analyze the potential functions of interacting proteins and corresponding signal pathways.

### Quantitative Real-Time PCR(qRT-PCR) Assays Gene Expression Level

We try to understand the impact of the interaction of BspJ with the NME2 and CKB proteins on the expression of these proteins. The pcDNA3.1-BspJ and pcDNA3.1 plasmids were transfected into HEK293T cells with Lipofectamine 3000 (Invitrogen, United States). After 24 h, add TRIzon Reagent (CWBIO, China) to lyse the cells, use Ultrapure RNA Kit (DNase I) (CWBIO, China) to extract cell total RNA, and then use HiFiScript cDNA Synthesis Kit (CWBIO, China) to reverse into cDNA. Using cDNA as a template, qRT-PCR was performed using the ABI QuantStudio^TM^ 5 (Thermo Fisher Scientific, United States) instrument and SYBR Green fluorescent DNA binding dye, which assays gene expression level of NME2 and CKB. The relative expression level normalization is for GAPDH. The sequences of primers were NME2-F: CCTGGGCTGGTGAAGTACATGAAC, NME2-R: TGGTGCCTGGCTTTGAATCTGC; CKB-F: CGACTTCAGA AGCGAGGCACAG, CKB-R: TCACTCCGTCCACCACCAT CTG and GAPDH-F: GGAGCGAGATCCCTCCAAA AT, GAPDH-R: GGCTGTTGTCATACTTCTCATGG. The amplification cycle steps are 95° for 30 s, 60° for 30 s, and 72° for 30 s. The 2^–ΔΔ*CT*^ approach was used to calculate the qRT-PCR data, and the values were normalized based on the expression level of housekeeping genes.

### Determining the Intracellular Survival of Brucella Abortus ΔBspJ

RAW264.7 cells were subcultured in 12-well plates. The cells were infected with *Brucella abortus* 2308, *Brucella abortus* ΔBspJ and *Brucella abortus* pBspJ in a MOI 1:100. After 60 min, add 2.5 μL of gentamicin (50 μg/mL) to each well for 50 min to kill any extracellular *Brucella*. Lysozyme (0.2%) was added to release the intracellular bacteria post-infected 12, 24, and 48 h, and the bacterial was diluted 10^–2^, 10^–3^, 10^–4^, and 10^–5^, and then coated on TSA. Incubate the bacteria at 37°C for 3 days and record the number of bacteria in the dish.

### Flow Cytometry to Detect Cell Apoptosis

Macrophage RAW264.7 were cultured in 12-well plates and the number reached 1.0 × 10^6^ cells/well. *Brucella abortus* strain 2308, *Brucella abortus* ΔBspJ and *Brucella abortus* pBspJ, respectively, infect macrophages at a MOI 100:1. Post-infected 24 h, discard the culture medium, add trypsin (without EDTA) (Biosharp, China) to digest the cells, and treat the cells according to the Annexin V-FITC/PI apoptosis detection kit (Vazyme, China) operating instructions. Then use BD FACSAria III (BD Biosciences, United States) to perform all flow cytometric analyses. The individual test was repeated three times. FlowJo software was used for data analysis.

### Data Analysis

The test data represent the results of three independent experiments. All data in this study are presented as means ± SEM. Statistical significance and correlation analyses were calculated with SPSS Statistics 23. SNK test, One-way ANOVA and student’s *t*-tests were used for comparisons with different groups. Values of ^∗^*p* < 0.05, ^∗∗^*p* < 0.01, and ^∗∗∗^*p* < 0.001 were considered to be statistically significant. GraphPad Prism software was used to construct the figures.

## Results

### Brucella Protein BspJ Can Be Translated in the Host Cell Nucleus

**FIGURE 1:**
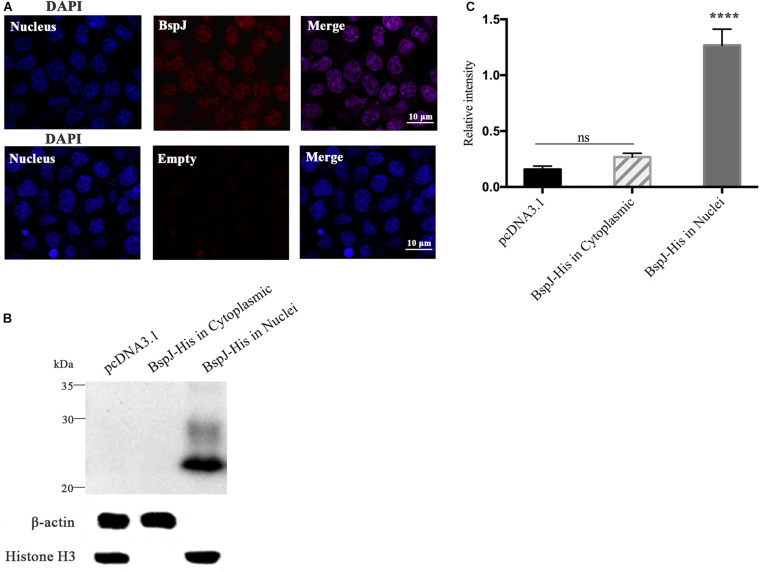
Identification of BspJ in the host HEK293T cell nucleus. **(A)** The pDsRed2-C1-BspJ and pDsRed2-C1(Empty) were transferred into HEK293T cells, and DMEM was replaced with 10% FBS DMEM after 6 h. After 24 h of culture, the cells were observed by confocal microscopy. **(B)** The pcDNA3.1-BspJ or pcDNA3.1 were transferred into HEK293T cells, and DMEM was replaced with 10% FBS DMEM after 6 h. After 48 h of culture, cellular proteins in the cytoplasmic or nuclei were collected for western blot analysis. **(C)** Relative intensity of BspJ were semi-quantified using ImageJ software. Data were shown as means ± SEM, ****p < 0.0001. The results in the photographs were obtained from three independent replicate experiments.

We studied the subcellular localization of the BspJ protein. After pDsRed2-C1-BspJ was transferred into HEK293T cells, DAPI staining of the nucleus and scanning with a confocal microscope revealed that the BspJ protein was mainly located in the host cell nucleus, while it was not found in the control pDsRed2-C1 (Empty) (Figure 1A), which initially confirming our conjecture. In the following western blotting experiment, pcDNA3.1-BspJ was transferred into HEK293T cells, and the presence of BspJ was detected in the nucleus but not in the cytoplasm or the empty vector control (Figure 1B), implying that BspJ can enter the host cell nucleus. The semi-quantitative results of BspJ relative intensity are shown as bar graphs in Figure 1C. Through bioinformatics analysis, we found that BspJ had no signal peptide (Supplementary Figure 2A) and no transmembrane structure (Supplementary Figure 2B), but it did have a nuclear localization signal (NLS) (Supplementary Figure 2C) and nuclear export signal (NES) (Supplementary Figure 2D). This bioinformatics results indicates that the BspJ protein could be secreted by *Brucella* into the host cell nucleus. In short, after multiple experiments, we found that BspJ can enter the host cell nucleus after being translated, and the protein will have a specific function.

### Screened Out HEK293T Cell Potential Proteins That Interact With BspJ

After verifying the expression of the BspJ protein in the host cell nucleus, we used the yeast two-hybrid method to screen for the interaction proteins of BspJ in the HEK293T cDNA library. BspJ contains 176 amino acids and has a size of 20.3 kDa. The pGBKT7-BspJ was transformed into yeast AH109 (*MAT*α) cells, and single clones that grew well on the DDO plate were chosen. There were white plaques on the DDO plate and sterile plaques on the QDO plate (Supplementary Figure 1B), that indicated that the BspJ protein was non-toxic and had no self-activating activity.

**FIGURE 2:**
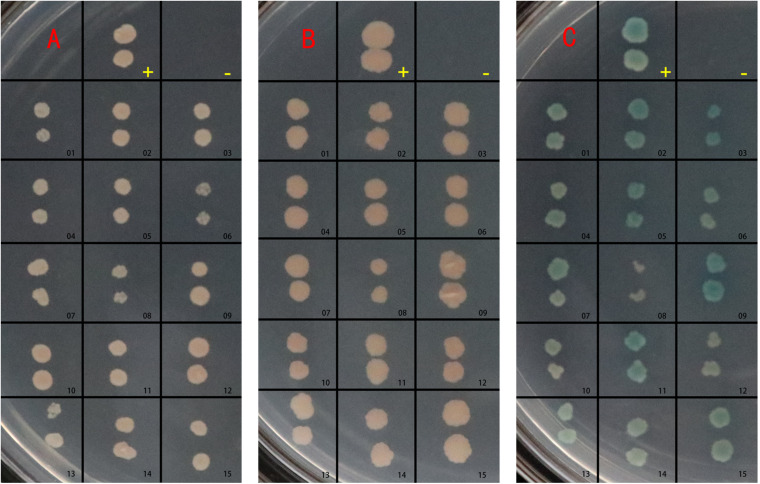
BspJ potential interaction protein gene transfer back verification test. -, Negative control group; pGBKT7/pGADT7. +, Positive control group; pGBKT7-53/pGADT7-T. Others, groups to be verified. **(A)** Growth on SD/–Leu/–Trp (DDO) medium. **(B)** Growth on SD/–Ade/–His/–Leu/–Trp (QDO) medium. **(C)** Growth on SD/–Ade/–His/–Leu/–Trp/X-α-gal (QDO/X) medium. The plasmid to be verified and pGBKT7-BspJ plasmid were co-transformed into AH109 yeast competent cells, coated with DDO, cultured for 3–5 days, and photographed. Then QDO and QDO/X were applied, and photographs were taken after 5–14 days. The results in the figure were obtained from three independent replicate experiments.

The pGBKT7-BspJ and pGADT7-cDNA plasmids were co-transferred into yeast cells and cultured. After screening for defective media, preliminary screening on DDO plates, and then database comparison with QDO selection and sequencing, we found that 15 histones interacted with BspJ. The reversion verification test found that after screening by DDO, QDO, and QDO/X auxotrophic plates, all 15 groups of proteins containing interaction signals with BspJ (Figure 2) Sequencing data and statistical results of potentially interacting proteins with BspJ showed in Supplementary Figures 3-5.

### Database Comparison and Analysis of Potential Interacting Proteins

We analyzed the nucleotide sequences of fifteen potential interacting proteins. After sequencing and database comparison, the repetitive signals were screened out, and four potential proteins were identified (Table 1). The four BspJ interacting proteins were *Homo sapiens* macrophage migration inhibitory factor (MIF), *Homo sapiens* NME/NM23 nucleoside diphosphate kinase 2 (NME2), *Homo sapiens* rib creatine kinase B (CKB), and *Homo sapiens* ribosomal protein L13 (RPL13).

**TABLE 1:** Database analysis of four potential BspJ interacting proteins.

Number	BLAST comparison prediction results	Sequence ID	Length
BspJ-a	*Homo sapiens* macrophage migration inhibitory factor (MIF), mRNA	NM_002415.1	348 bp
BspJ-b	*Homo sapiens* NME/NM23 nucleoside diphosphate kinase 2 (NME2), transcript variant 1, mRNA	NM_002512.3	459 bp
BspJ-c	*Homo sapiens* creatine kinase B (CKB), transcript variant X1, mRNA	XM_017020951.1	1218 bp
BspJ-d	*Homo sapiens* ribosomal protein L13 (RPL13), transcript variant 1, mRNA	NM_000977.3	636 bp

### CO-IP Verifies Protein-Protein Interactions

**FIGURE 3:**
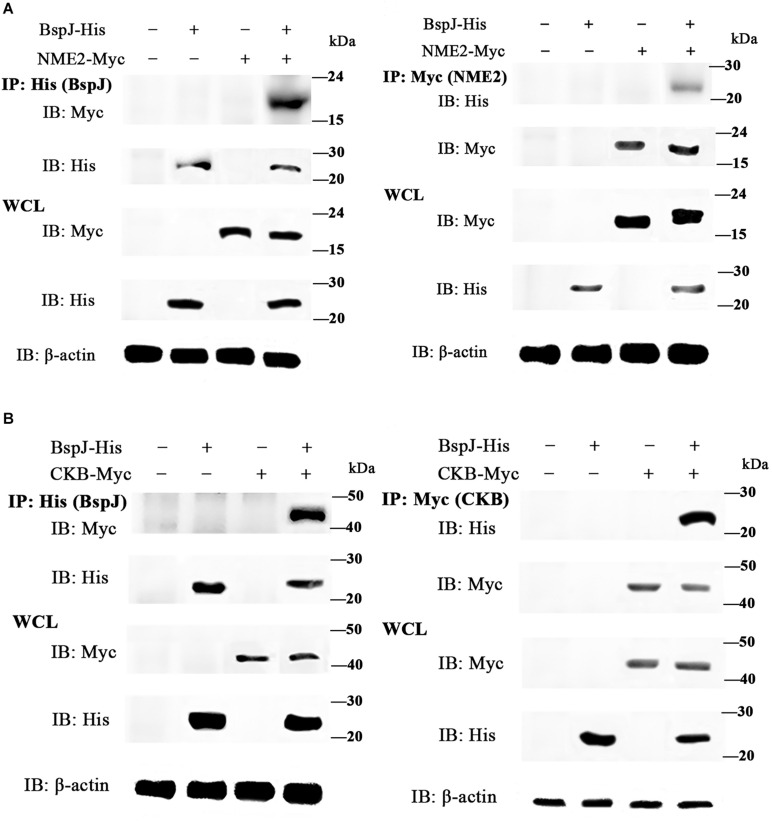
BspJ interacts directly with NME2 and CKB. **(A)** BspJ interacts with NME2. **(B)** BspJ interacts with CKB. BspJ-His, NME2-Myc, CKB-Myc or empty vectors were transfected into HEK293T cells, which were then used either alone or in various combinations. After 48 h, cells were harvested for incubation with anti-His antibodies (left) or anti-Myc antibodies (right), and G-Agarose beads were used for co-immunoprecipitation. Immunoblots were conducted with the indicated antibodies. Relative intensity for BspJ-His and NME2-Myc, BspJ-His and CKB-Myc were semi-quantified using ImageJ software and presenting as bar graphs in Supplementary Figure 6. Images are representative from three independent experiments.

We used a yeast two-hybrid assay to screen for potential interaction proteins with BspJ; these were MIF, NME2, CKB, and RPL13. Next, the interactions between the proteins and BspJ were verified by CO-IP. After both of pcDNA3.1-MIF/NME2/CKB/RPL13 and pcDNA3.1-BspJ was transferred into HEK293T cells, whole cell lysate (WCL) western blot analysis detected the presence of four proteins. However, in the co-immunoprecipitation test, we only detected the presence of NME2 (Figure 3A and Supplementary Figures 6A,B) and CKB (Figure 3B and Supplementary Figures 6C,D) using the His and Myc Tag antibodies to pull down the cell protein solution, indicating that NME2 and CKB did indeed interact with BspJ (Figure 3 and Supplementary Figure 6).

### GO Analysis of BspJ-Interacting Proteins

**FIGURE 4:**
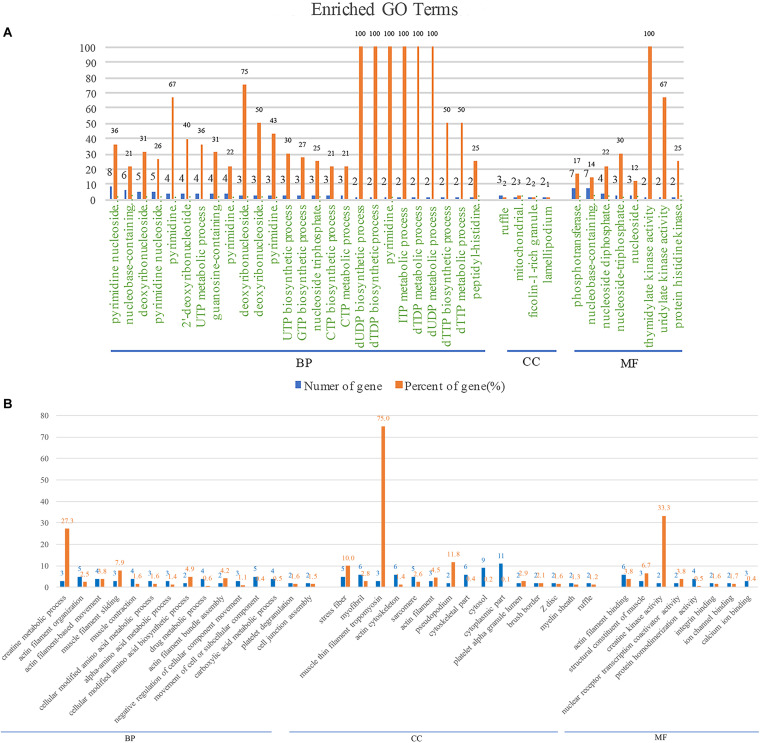
NME2 and CKB GO terms analysis. BP, biological process; CC, cellular component; MF, molecular function. **(A)** NME2 protein GO terms analysis. **(B)** CKB protein GO terms analysis. A represents part of GO analysis items, see Supplementary Figure 7 for details.

In order to explore the biological processes involved in BspJ-interacting proteins (NME2 and CKB), we performed a GO enrichment analysis (Figure 4 and Supplementary Figure 7). NME2 is mainly involved in the synthesis and metabolism of cellular energy components. Among these, the synthesis and metabolism of dTDP, dUDP, dTTP, UTP, and deoxynucleoside phosphate occupy major positions. In addition, NME2 also participates in the activation process of some enzymes, such as nucleoside phosphokinase, thymidylate kinase and uridylate kinase, all of which represent biological processes and molecular functions in energy metabolism pathways (Figure 4A and Supplementary Figure 7). CKB is mainly related to the biosynthetic metabolic processes related to actin, creatine, and myofilaments. In the Cellular Component category, CKB mainly includes components such as stress fiber, tropomyosin, and pseudopodium. Otherwise, CKB possesses the activity of creatine kinase and nuclear receptor transcription coactivator and exerts molecular functions such as the composition and binding of actin filaments (Figure 4B). In general, NME2 is involved in cell energy metabolism and synthesis, while CKB is involved in biological processes such as cell movement activities, but these are all related to the body’s energy pathways.

### Pathway Analysis of BspJ-Interacting Proteins

**FIGURE 5:**
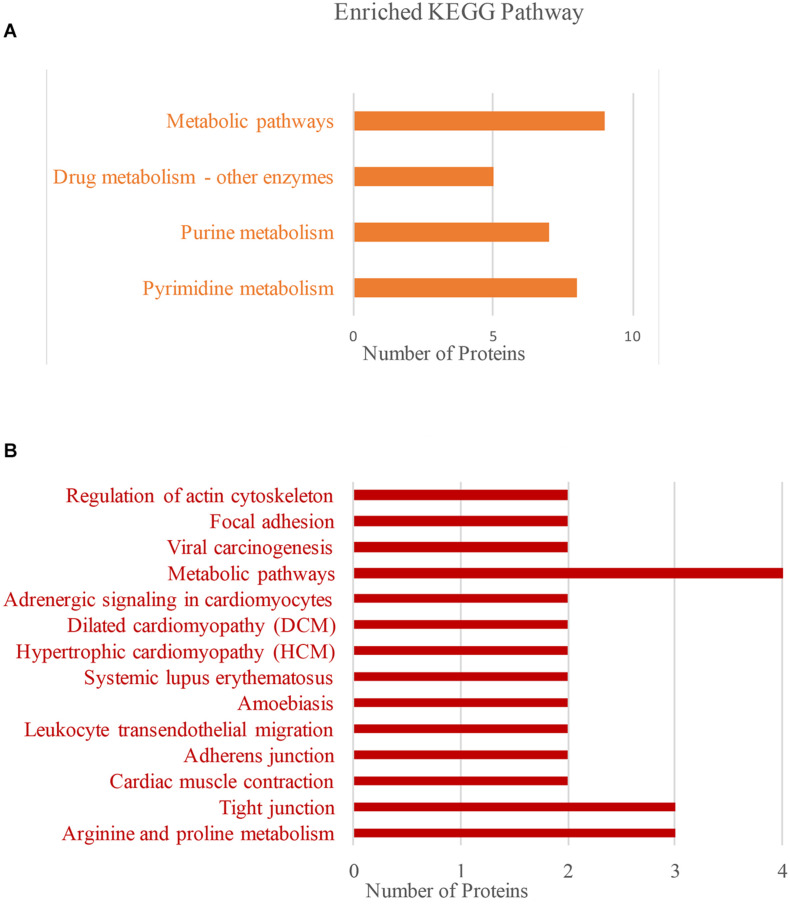
NME2 and CKB KEGG pathway analysis. **(A)** NME2 protein KEGG pathway analysis. **(B)** CKB protein KEGG pathway analysis.

Next, we used KEGG to analyze the main pathways of BspJ-interacting proteins (Figure 5). NME2 is mainly involved in the cell metabolism pathways such as pyrimidine metabolism, purine metabolism, and drug metabolism involving other enzymes (Figure 5A). The CKB KEGG analysis also identified cell metabolism pathways (arginine and proline metabolism), and also included some connection regulation effects (tight junction, drug-enzymes metabolism, and actin cytoskeleton regulation). It is worth noting that the pathways are also involved in the occurrence and signal transduction of some diseases, such as amoebiasis, systemic lupus erythematosus, cardiomyocyte disease, hypertrophic cardiomyopathy (HCM), dilated cardiomyopathy (DCM), and adrenergic signaling in cardiomyocytes (Figure 5B). In summary, NME2 and CKB are both involved in the cell metabolism pathways, a finding that is somewhat related and similar to the energy metabolism and synthesis shown in the GO analysis. In addition, CKB is also involved in the occurrence of some diseases, for example HCM, DCM, and systemic lupus erythematosus.

### Protein-Protein Interaction (PPI) Pathway Analysis and Prediction

**FIGURE 6:**
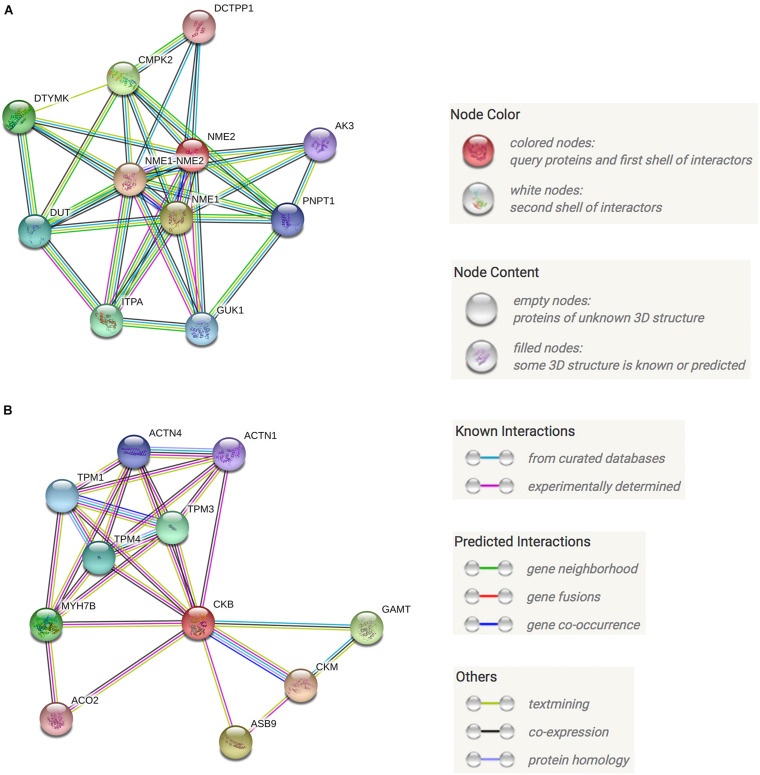
NME2 and CKB KEGG PPI analysis. **(A)** NME2 protein PPI analysis. **(B)** CKB protein PPI analysis.

Protein-protein interaction (PPI) networks can better reflect the interactions between proteins. Through PPI pathway analysis, we predicted and analyzed the main known and potential proteins interacting with NME2 and CKB (Figure 6). The identification of these proteins will help analyze the function and action network of BspJ-interacting proteins. After analysis and searching, NME2 and CKB did not appear to have a common interacting protein (Figures 6A,B), but this does not prevent some of their similar mechanisms of action (i.e., participation in cell metabolism).

### BspJ Decreased the Expression Level of the Interacting Protein NME2 and CKB

**FIGURE 7:**
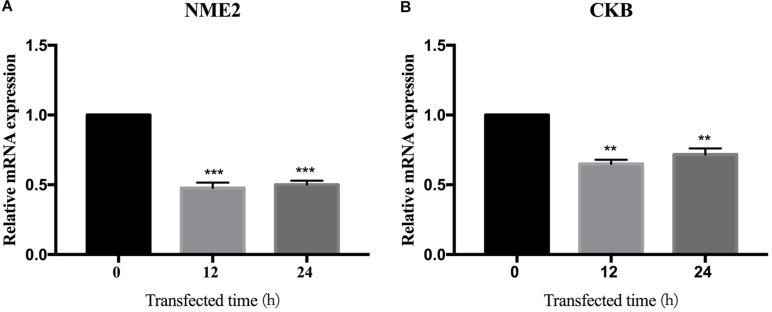
BspJ inhibited the expression of interacting proteins NME2 and CKB. After pcDNA3.1-BspJ was transferred into HEK293T cells, cell samples were harvested at 12 and 24 h, respectively, and the expression of NME2 **(A)** and CKB **(B)** was detected. Results represent the results of three independent tests. Data were shown as means ± SEM, *p < 0.05, **p < 0.01, and ***p < 0.001.

We found the interaction between BspJ and NME2 and CKB, and bioinformatically analyzed NME2 and CKB. Next, we tried to find out whether BspJ could affect the expression of NME2 and CKB. The mRNA test results showed that BspJ significantly reduced the expression levels of NME2 and CKB (Figure 7), and there was no difference in this inhibition after BspJ was transfected into host cells for 12 h and 24 h (Figure 7). It shows that this inhibition is not time-dependent. In addition, it can be found that, compared with NME2 (Figure 7A), the inhibitory effect of BspJ on CKB is weaker (Figure 7B).

### BspJ Enhances the Intracellular Survival of Brucella and Inhibits the Apoptosis of Macrophages

**FIGURE 8:**
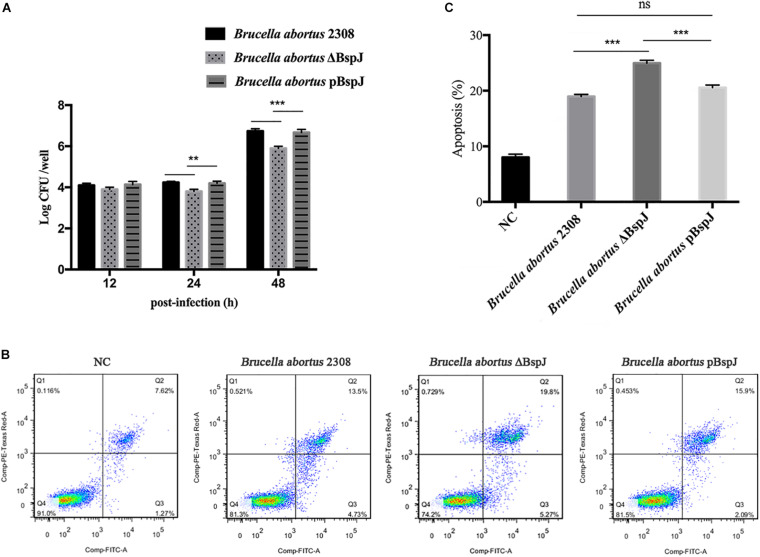
BspJ enhances the intracellular survival of *Brucella* and inhibits the apoptosis of macrophages. **(A)** BspJ enhances the intracellular survival of *Brucella*. The parental strain, deletion strain and complementary strain to infect cells RAW264.7, post-infected 12, 24, and 48 h, CFU count of intracellular bacteria was performed. **(B)** BspJ intensified the apoptotic efficiency of macrophage RAW264.7. After the parent strain, deletion strain and complementary strain were infected with RAW264.7 for 24 h, the cells were collected to perform flow cytometric assay. **(C)** Statistical analysis of apoptosis rate of macrophage RAW264.7. All data were shown as means ± SEM from three independent tests, **p < 0.01 and ***p < 0.001. ns, no significant difference.

After infected macrophages, *Brucella abortus* 2308, *Brucella abortus* ΔBspJ and *Brucella abortus* pBspJ showed varying degrees of intracellular survival differences (Figure 8A). At post-infected 12 h, there was no difference in intracellular survival among the parental strain, the deletion strain and the complementary strain. However, at post-infected 24 h, the intracellular survival number of *Brucella abortus* ΔBspJ was significantly lower than that of *Brucella abortus* 2308 and *Brucella abortus* pBspJ, and the difference was more significant at post-infected 48 h (Figure 8A). In addition, the intracellular viability of *Brucella abortus* 2308 and *Brucella abortus* pBspJ is always the same (Figure 8A), which shows that *Brucella abortus* pBspJ compensates for the growth defect caused by the lack of BspJ. These results indicate that the deletion of BspJ can reduce the intracellular viability of *Brucella*, and may play an important role as a potential virulence factor of *Brucella*.

We used the parental strain, the deletion strain and the complementary strain to infect cells RAW264.7 24 h later, and then measured the apoptosis rate of macrophages using flow cytometry (Figures 8B,C). As the results show, the apoptotic rate of macrophages infected by *Brucella abortus* ΔBspJ was significantly higher than that of *Brucella abortus* 2308 group and *Brucella abortus* pBspJ group (*p* < 0.001). However, there was almost no difference between *Brucella abortus* 2308 group and *Brucella abortus* pBspJ group (Figures 8B,C). These results suggest that BspJ protein plays an important role in inhibiting host cell apoptosis.

**FIGURE 9:**
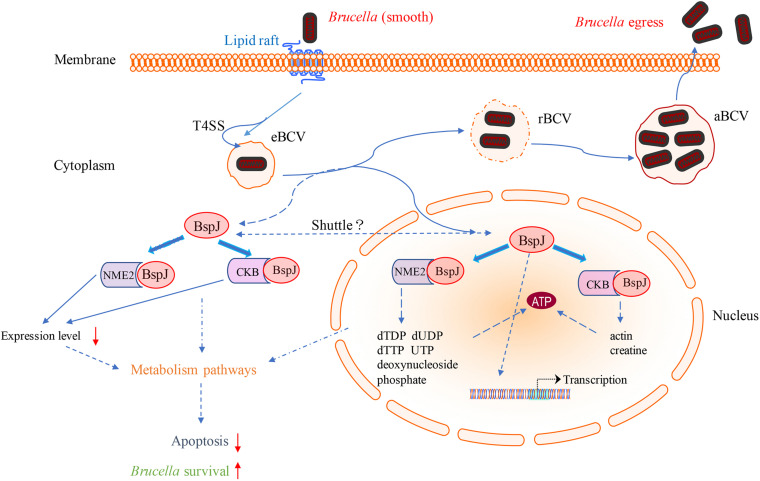
Diagram of the role of BspJ in host cells. The solid line represents the known results, and the dashed line represents the potential results.

Combining all the above results, we finally mapped the regulatory processes of BspJ and BspJ-interacting proteins involved in host cells in order to more clearly illustrate the role and functional mechanism of BspJ (Figure 9).

## Discussion

Many Gram-negative bacteria, such as *Legionella pneumophila*, *Brucella*, *Burkholderia*, *Helicobacter pylori*, and *Bartonella*, use T4SS to secrete effector proteins into host cells, and this is an important part of their pathogenic process (Pan et al., 2008; Chen et al., 2010; Adi et al., 2015). BspJ is a newly discovered *Brucella* secretory protein, and its translocation pathway needs to be further studied. The TEM1 reporter fusion protein test showed that BspJ was a protein dependent on T4SS secretion, while the CyaA reporter fusion protein test showed that the CyaA reporter fusion BspJ protein C-terminal or N-terminal T4SS secretion dependence was inconsistent (Myeni et al., 2013). These results indicate that the position of the reporter may interfere with the translocation signal and affect the secretion of BspJ. Another possibility is that the BspJ protein of *Brucella* may be secreted through a mixed translocation system instead of relying solely on T4SS (Myeni et al., 2013). Studies have shown that *Salmonella* SptP (Lee et al., 2004) and *Yersinia* YplA (Young and Young, 2002) can secrete effectors into host cells through the type III secretion system (T3SS) and flagellar export system. *Brucella* has a full set of genes coding for proteins that are assembled into functional flagella, but not all coding genes are expressed (Delvecchio et al., 2002). In some virulent strains of *Brucella*, fully formed flagella can be observed (Sidhu-Muñoz et al., 2020). Therefore, T4SS and flagella may play a regulatory role together in *Brucella* (Delrue et al., 2005). Some of the secretory proteins of *Brucella* may be targeted by the flagella export pathway and translocated to host cells, but this pathway remains to be verified.

In our research results, whether using confocal microscopy or western blotting, it was confirmed that BspJ was secreted into the host cell nucleus (Figure 1). As mentioned in the Introduction, there are indeed many *Brucella* effector proteins, and they participate in a variety of host regulatory pathways (e.g., apoptosis, autophagy, and inflammation) (Celli, 2019). However, to our knowledge, BspJ is the first *Brucella* nucleomodulin to be described. It was reported that LntA, a secreted virulence factor of *Listeria monocytogenes*, can stimulate the innate immune response by inhibiting the chromatin-related repressor BAHD1 (Lebreton et al., 2014). The obligate intracellular parasitic bacterium *Ehrlichia chaffeensis* effector TRP32 enters the host cell nucleus through a non-classical translocation mechanism, binds to host DNA, and changes host gene transcription to regulate various host cell processes (including cell differentiation and proliferation), a mechanism that involves the phosphorylation of Y179 located in the C-terminal trityl tyrosine motif (Farris et al., 2016, 2017). TRP120, another effector of *Ehrlichia chaffeensis*, not only plays a role in protein binding and internalization but also translocates to the host cell nucleus, and it is thus considered to be a transcription factor that regulates gene expression. However, the mechanism by which TRP120 binds to DNA and regulates gene expression is still elusive (Klema et al., 2018). In general, although we have discovered the effector protein BspJ of *Brucella* in the nucleus consistent with reported earlier (Myeni et al., 2013), the mechanism of its entry into the nucleus is still unknown, and the effector function after entering the nucleus is worth exploring.

NME2, also known as NDPK B, belongs to the NME family of genes encoding nucleoside diphosphate kinases (NDPKs), of which NME1/NME2 has been the most studied (Gong et al., 2020), and we have also found a close relationship between NME1 and NME2 (Figure 6A). According to previous reports, NME1 and NME2 are located in different positions in the cell such as the cytosol, the membrane binding compartment (plasma membrane, endoplasmic reticulum, or cytoplasmic vesicle), and the nucleus (Boissan and Lacombe, 2011). In addition, NME2 is believed to be responsible for at least 80% of NDPK activity (Boissan et al., 2005). NME2 promotes the development of erythropoiesis, because the iron transport receptor TfR1 in the red blood cells of Nme1/Nme2-deficient mice is down-regulated, characterized by hypoplasia, severe anemia, and perinatal death (Postel et al., 2009). It is worth noting that NME2 has the functions of metastasis inhibition and regulation of cell apoptosis. The depletion of mouse NME2 will increase the effectiveness of ERK signaling and enhance the proliferation of fibroblasts (Lee et al., 2009). High levels of NME2 cooperate with other metastasis inhibitors (such as TIP30) to enhance cell apoptosis, which involves the effect of inducing apoptosis related genes (Thakur et al., 2011). It has recently been confirmed that NME2 can mediate the transcription of anti-apoptotic miRNA (miR-100) and anti-apoptotic protein coding genes (RIPK1, STARD5, and LIMS1) (Gong et al., 2020). Unlike many pathogens that are released after the death of host cells, *Brucella* will prevent the death of host cells in order to maintain its intracellular living environment (Gross et al., 2000; Cui et al., 2014). In our results, nucleomodulin BspJ plays an important role in inhibiting apoptosis of macrophages (Figures 8B,C), but the specific mechanism is unclear. It is worth noting that compared with wild strain, the cell viability of BspJ gene-deficient strain is significantly reduced (Figure 8A), which suggests that BspJ may also play an important role as a virulence protein in promoting the intracellular survival of *Brucella*. Therefore, it cannot be ruled out that BspJ, the secretory protein of *Brucella*, mediates the process of host cell apoptosis by binding to the anti-apoptotic factor NME2 and thereby satisfying the long-term intracellular survival of *Brucella*.

Creatine kinase (CK), including muscle type CK (CKM) and brain type CK (CKB), can transfer high-energy phosphate from phosphocreatine (PCr) to adenosine diphosphate (ADP) to regulate the regeneration of ATP (Wallimann, 1994). CK recruited adaptors directly or indirectly interact with various ATPases and participate in many physiological processes (Chen et al., 2011). Knockdown of CKB can affect cell energy and metabolic status, change signal transduction pathways, and further affect cell proliferation. Studies have found that the phosphorylation of p21 and Akt is inhibited by CKB. CKB knockdown can up-regulate the expression of p21. P21 is the main member of the Cip/Kip family of Cdk inhibitors, and it has a negative regulatory effect on cell proliferation and cell cycle progression (Coqueret, 2003). CKB knockdown also caused tumor cell cycle arrest in the G2 phase, resulting in inactivation of Akt and increased apoptosis of Skov3 cells (Lincet et al., 2000), and more significant effects were observed under hypoxia and hypoglycemia conditions (Li et al., 2013). The anti-apoptotic effect of Akt is consistent with its function in energy metabolism (Robey and Hay, 2009).

It has recently been reported that miR-483-mediated CKB silencing disrupts cellular ATP homeostasis and promotes endoplasmic reticulum stress-induced apoptosis (Hiramatsu et al., 2020). Kapetanovich et al. (2004) proposed a potential role of NME2 in cytoplasmic coat protein complex II (COPII)-mediated endoplasmic reticulum transport and found that knocking out NME2 would reduce COPII assembly and reduce endoplasmic reticulum output. *Brucella* infection of host cells does cause endoplasmic reticulum stress and induces unfolded protein response (UPR) in macrophages or HeLa cells (de Jong et al., 2013; Smith et al., 2013; Taguchi et al., 2015). UPR is indispensable for *Brucella* replication and survival (Qin et al., 2008). In summary, both NME2 and CKB are related to energy metabolism pathways, a result that has also been confirmed by our pathway analysis (Figure 5), and both of these proteins have the effect of inhibiting cell apoptosis. Inhibiting host cell apoptosis is an important step for *Brucella* to complete its intracellular circulation (Celli, 2019). The secreted protein BspJ of *Brucella* does interact with NME2 and CKB (Figure 3). Meanwhile, BspJ inhibits the expression of NME2 and CKB (Figure 7), which may affect the original biological functions of NME2 and CKB, but this requires more research to verify this conjecture. From this we have concluded that after BspJ is secreted by *Brucella*, it interacts with NME2 and CKB through a series of unknown mechanisms, directly or indirectly inhibiting the process of host cell apoptosis and completing the intracellular cycle of *Brucella* (Figure 9), but this has yet to be verified.

Our research has found that BspJ interacts with NES and NLS (Supplementary Figures 2C,D), indicating that BspJ may have a nuclear-cytoplasmic shuttling process and may exist in other organelles outside the nucleus (Figure 9). The nuclear-cytoplasmic shuttling process has been studied in many viral proteins, including influenza virus (Boulo et al., 2007), human hepatitis B virus (Li et al., 2010), and swine fever virus (Li et al., 2014). The viral proteins mainly act as carriers in signal transmission between the nucleus and the cytoplasm. It is known that NME2 and CKB exist in the nucleus and cytoplasm (Boissan and Lacombe, 2011; Ju et al., 2012), and BspJ interacts with both proteins, but in our study BspJ was detected in the nucleus of the host cell and not in the cytoplasm (Figure 1). This may be because BspJ is rarely present in the cytoplasm. In addition, the His tag may also affect the expression and shuttle process of BspJ (Myeni et al., 2013). Interestingly, when detecting the expression of BspJ in host cells, we find two BspJ expressed proteins (Figure 1B). This indicates that there may be processing and modification of BspJ expression in the host cell nucleus, which has been reported in the secretion of Ats-1 protein of *Anaplasma phagocytophilum* into mitochondria (Niu et al., 2010). Moreover, the influence of His tag on protein expression cannot be ruled out (Myeni et al., 2013).

## Conclusion

In our study, we defined the first *Brucella* nucleomodulin protein (BspJ) that enters to the host cell nucleus, and screening identified the host proteins NME2 and CKB that interact with BspJ. We found that the transcription of BspJ inhibits the expression of NME2 and CKB. In addition, the deletion of BspJ aggravated the apoptosis of macrophages and reduced the intracellular viability of *Brucella*. Finally, we hypothesized that nucleomodulin BspJ may mediate cell energy metabolism pathways to directly or indirectly regulate the process of host cell apoptosis in order to complete the intracellular circulation of *Brucella*. However, this hypothesis needs more data for verification.

## Data Availability Statement

The raw data supporting the conclusions of this article will be made available by the authors, without undue reservation.

## Author Contributions

ZM and YW designed the study. ZM, RL, RH and XD were responsible for the provision, integration, and writing of the article. YW and CC reviewed the article, and the other authors provided help during the experimental process. All authors contributed to the article and approved the submitted version.

## Conflict of Interest

The authors declare that the research was conducted in the absence of any commercial or financial relationships that could be construed as a potential conflict of interest.

## Footnotes

1 https://blast.ncbi.nlm.nih.gov

2 www.cbs.dtu.dk/services/TMHMM/

3 http://www.cbs.dtu.dk/services/SignalP/

4 http://nls-mapper.iab.keio.ac.jp/cgi-bin/NLS_Mapper_form.cgi

5 http://www.cbs.dtu.dk/services/NetNES/

6 https://string-db.org/cgi/input.pl

## Supplementary Material

Supplementary Figure 1 **(A)** The detailed PCR parameters of identify the recombination rate and insert length of HEK293T cDNA library. **(B)** BspJ gene self-activation and toxicity detection. -. pGBKT7/pGADT7 negative control. +. pGBKT7-53/pGADT7-T positive control. (a-f) BspJ/pGADT7 test group. Growth on SD/–Ade/–His/–Leu/–Trp (QDO) medium. **(C)** Identifying the insert length in HEK293T cDNA library. N, Blank control. M, DNA marker. 1-24, PCR product of the sample. The results in the figure were obtained from three independent replicate experiments. Images are representative from three independent experiments.

Supplementary Figure 2 **(A)** The result of BspJ signal peptide prediction. **(B)** The result of BspJ transmembrane structure prediction. **(C)** The result of BspJ protein nuclear localization signal (NLS) prediction. **(D)** The result of BspJ protein nuclear export signal (NES) prediction.

Supplementary Figure 3 Sequencing data and statistical results of 15 potentially interacting proteins with BspJ.

Supplementary Figure 4 Sequencing data and statistical results of 15 potentially interacting proteins with BspJ.

Supplementary Figure 5 Sequencing data and statistical results of 15 potentially interacting proteins with BspJ.

Supplementary Figure 6 Relative intensity for BspJ-His and NME2-Myc, BspJ-His and CKB-Myc were semi-quantified using ImageJ software. Images are representative from three independent experiments.

Supplementary Figure 7 The details GO analysis items of NME2.

## References

[B1] Adi P. J. Bhashyam S. S. Konidala K. K. Bhaskar M. (2015). Complete genome-wide screening and subtractive genomic approach revealed new virulence factors, potential drug targets against bio-war pathogen *Brucella melitensis* 16M. *Drug Design Dev. Ther.* 2015 1691–1706. 10.2147/dddt.s76948 25834405 PMC4371898

[B2] Alaidarous M. Ve T. Casey L. W. Valkov E. Ericsson D. J. Ullah M. O. (2014). Mechanism of bacterial interference with TLR4 signaling by Brucella Toll/interleukin-1 receptor domain-containing protein TcpB. *J. Biol. Chem.* 289 654–668. 10.1074/jbc.m113.523274 24265315 PMC3887194

[B3] Atluri V. L. Xavier M. N. De Jong M. F. Den Hartigh A. B. Tsolis R. M. (2011). Interactions of the human pathogenic *Brucella* species with their hosts. *Annu. Rev. Microbiol.* 65 523–541. 21939378 10.1146/annurev-micro-090110-102905 PMC13363517

[B4] Avilacalderon E. D. Lopezmerino A. Sriranganathan N. Boyle S. M. Contrerasrodriguez A. (2013). A history of the development of Brucella vaccines. *BioMed Res. Int.* 2013:743509. 10.1155/2013/743509 PMC3686056 23862154

[B5] Boissan M. Lacombe M. (2011). Learning about the functions of NME/NM23 : lessons from knockout mice to silencing strategies. *Naunyn-schmiedebergs Arch. Pharmacol.* 384 421–431. 10.1007/s00210-011-0649-3 21562815

[B6] Boissan M. Wendum D. Arnaud-Dabernat S. Munier A. Debray M. Lascu I. (2005). Increased lung metastasis in transgenic NM23-Null/SV40 mice with hepatocellular carcinoma. *JNCI J. Natl. Cancer Instit*. 97 836–845. 10.1093/jnci/dji143 15928304

[B7] Boschiroli M. L. Ouahrani-Bettache S. Foulongne V. Michaux-Charachon S. Bourg G. Allardet-Servent A. (2002). The Brucella suis virB operon is induced intracellularly in macrophages. *Proc. Natl. Acad. Sci. U.S.A.* 99 1544–1549. 10.1073/pnas.032514299 11830669 PMC122227

[B8] Boulo S. Akarsu H. Ruigrok R. W. H. Baudin F. (2007). Nuclear traffic of influenza virus proteins and ribonucleoprotein complexes. *Virus Res.* 124 12–21. 10.1016/j.virusres.2006.09.013 17081640

[B9] Celli J. (2015). The changing nature of the B rucella-containing vacuole. *Cell. Microbiol.* 17 951–958. 10.1111/cmi.12452 25916795 PMC4478208

[B10] Celli J. (2019). Brucella the intracellular life cycle of spp. *Microbiol. Spectr.* 7. 10.1128/microbiolspec.BAI-0006-2019 30848234 PMC6448592

[B11] Celli J. de Chastellier C. Franchini D.-M. Pizarro-Cerda J. Moreno E. Gorvel J.-P. (2003). Brucella evades macrophage killing via VirB-dependent sustained interactions with the endoplasmic reticulum. *J. Exp. Med.* 198 545–556. 10.1084/jem.20030088 12925673 PMC2194179

[B12] Chen C. Banga S. Mertens K. Weber M. M. Gorbaslieva I. Tan Y. (2010). Large-scale identification and translocation of type IV secretion substrates by *Coxiella burnetii*. *Proc. Natl. Acad. Sci. U.S. A.* 107 21755–21760. 10.1073/pnas.1010485107 21098666 PMC3003115

[B13] Chen Z. Zhao T. Li J. Gao Y. S. Meng F. G. Yan Y. (2011). Slow skeletal muscle myosin-binding protein-C (MyBPC1) mediates recruitment of muscle-type creatine kinase (CK) to myosin. *Biochem. J.* 436 437–445. 10.1042/bj20102007 21426302

[B14] Comerci D. J. Martínez-Lorenzo M. J. Sieira R. Gorvel J. P. Ugalde R. A. (2001). Essential role of the VirB machinery in the maturation of the Brucella abortus-containing vacuole. *Cell. Microbiol.* 3 159–168. 10.1046/j.1462-5822.2001.00102.x 11260139

[B15] Coqueret O. (2003). New roles for p21 and p27 cell-cycle inhibitors: a function for each cell compartment? *Trends Cell Biol.* 13 65–70. 10.1016/s0962-8924(02)00043-0 12559756

[B16] Cui G. Wei P. Zhao Y. Guan Z. Yang L. Sun W. (2014). Brucella infection inhibits macrophages apoptosis via Nedd4-dependent degradation of calpain2. *Vet. Microbiol.* 174 195–205. 10.1016/j.vetmic.2014.08.033 25258171

[B17] de Barsy M. Jamet A. Filopon D. Nicolas C. Laloux G. Rual J. F. (2011). Identification of a *Brucella* spp. secreted effector specifically interacting with human small GTPase Rab2. *Cell. Microbiol.* 13 1044–1058. 10.1111/j.1462-5822.2011.01601.x 21501366

[B18] de Jong M. F. Starr T. Winter M. G. den Hartigh A. B. Child R. Knodler L. A. (2013). Sensing of bacterial type IV secretion via the unfolded protein response. *mBio* 4:e00418-12. 10.1128/mBio.00418-12 PMC3624511 23422410

[B19] Delrue R. Deschamps C. Leonard S. Nijskens C. Danese I. Schaus J. (2005). A quorum-sensing regulator controls expression of both the type IV secretion system and the flagellar apparatus of *Brucella melitensis*. *Cell. Microbiol.* 7 1151–1161. 10.1111/j.1462-5822.2005.00543.x 16008582

[B20] Delrue R. M. Martinez-Lorenzo M. Lestrate P. Danese I. Bielarz V. Mertens P. (2001). Identification of *Brucella* spp. genes involved in intracellular trafficking. *Cell. Microbiol.* 3 487–497. 10.1046/j.1462-5822.2001.00131.x 11437834

[B21] Delvecchio V. G. Kapatral V. Redkar R. Patra G. Mujer C. V. Los T. (2002). The genome sequence of the facultative intracellular pathogen *Brucella melitensis*. *Proc. Natl. Acad. Sci. U. S. A.* 99 443–448. 11756688 10.1073/pnas.221575398 PMC117579

[B22] Farris T. Dunphy P. Zhu B. Kibler C. McBride J. (2016). *Ehrlichia chaffeensis* TRP32 is a nucleomodulin that directly regulates expression of host genes governing differentiation and proliferation. *Infect. Immun.* 84 3182–3194. 10.1128/iai.00657-16 27572329 PMC5067751

[B23] Farris T. Zhu B. Wang J. McBride J. (2017). *Ehrlichia chaffeensis* TRP32 nucleomodulin function and localization is regulated by NEDD4L-mediated ubiquitination. *Front. Cell. Infect. Microbiol.* 7:534. 10.3389/fcimb.2017.00534 29376035 PMC5768648

[B24] Gong Y. Yang G. Wang Q. Wang Y. Zhang X. (2020). NME2 is a master suppressor of apoptosis in gastric cancer cells via transcriptional regulation of miR-100 and other survival factors. *Mol. Cancer Res.* 18 287–299. 10.1158/1541-7786.mcr-19-0612 31694930

[B25] Green E. R. Mecsas J. (2016). Bacterial secretion systems–an overview. *Microbiol. Spectr.* 4 215–239. 10.1128/microbiolspec.VMBF-0012-2015 PMC4804464 26999395

[B26] Gross A. Terraza A. Ouahrani-Bettache S. Liautard J.-P. Dornand J. (2000). In vitro *Brucella suis* infection prevents the programmed cell death of human monocytic cells. *Infect. Immun.* 68 342–351. 10.1128/iai.68.1.342-351.2000 10603407 PMC97140

[B27] Hiramatsu N. Chiang K. Aivati C. Rodvold J. J. Lee J. Han J. (2020). PERK-mediated induction of microRNA-483 disrupts cellular ATP homeostasis during the unfolded protein response. *J. Biol. Chem.* 295 237–249. 10.1074/jbc.ra119.008336 31792031 PMC6952592

[B28] Jakka P. Namani S. Murugan S. Rai N. Radhakrishnan G. (2017). The Brucella effector protein TcpB induces degradation of inflammatory caspases and thereby subverts non-canonical inflammasome activation in macrophages. *J. Biol. Chem.* 292 20613–20627. 10.1074/jbc.m117.815878 29061850 PMC5733597

[B29] Ju T. C. Lin Y. S. Chern Y. (2012). Energy dysfunction in Huntington’s disease: insights from PGC-1α, AMPK, and CKB. *Cell. Mol. Life Ences* 69 4107–4120. 10.1007/s00018-012-1025-2 22627493 PMC11115139

[B30] Juhas M. Crook D. W. Hood D. W. (2008). Type IV secretion systems: tools of bacterial horizontal gene transfer and virulence. *Cell. Microbiol.* 10 2377–2386. 10.1111/j.1462-5822.2008.01187.x 18549454 PMC2688673

[B31] Kapetanovich L. Baughman C. Lee T. H. (2004). Nm23H2 facilitates coat protein complex II assembly and endoplasmic reticulum export in mammalian cells. *Mol. Biol. Cell* 16 835–848. 10.1091/mbc.e04-09-0785 15591128 PMC545915

[B32] Klema V. J. Sepuru K. M. Fullbrunn N. Farris T. R. Dunphy P. S. Mcbride J. W. (2018). Ehrlichia chaffeensis TRP120 nucleomodulin binds DNA with disordered tandem repeat domain. *PLoS One* 13:e0194891. 10.1371/journal.pone.0194891 29641592 PMC5895000

[B33] Lebreton A. Job V. Ragon M. Le Monnier A. Dessen A. Cossart P. (2014). Structural basis for the inhibition of the chromatin repressor BAHD1 by the bacterial nucleomodulin LntA. *mBio* 5:e00775-13. 10.1128/mBio.00775-13 24449750 PMC3903274

[B34] Lee M. C. S. Miller E. A. Goldberg J. Orci L. Schekman R. (2004). Bi-directional protein transport between the er and golgi. *Annu. Rev. Cell Dev. Biol.* 20 87–123. 10.1146/annurev.cellbio.20.010403.105307 15473836

[B35] Lee M. Y. Jeong W. J. Oh J. Choi K. (2009). NM23H2 inhibits EGF- and Ras-induced proliferation of NIH3T3 cells by blocking the ERK pathway. *Cancer Lett.* 275 221–226. 10.1016/j.canlet.2008.10.018 19022560

[B36] Li H. C. Huang E. Y. Su P. Y. Wu S. Y. Shih C. (2010). Nuclear export and import of human Hepatitis B virus capsid protein and particles. *PLoS Pathog.* 6:e1001162. 10.1371/journal.ppat.1001162 21060813 PMC2965763

[B37] Li X. Chen X. Ou W. Zhang Q. Lv Z. Zhan Y. (2013). Knockdown of creatine kinase B inhibits ovarian cancer progression by decreasing glycolysis. *Int. J. Biochem. Cell Biol.* 45 979–986. 10.1016/j.biocel.2013.02.003 23416112

[B38] Li Y. Shen L. Sun Y. Wang X. Li C. Huang J. (2014). Effects of the nuclear localization of the N(pro) protein of classical swine fever virus on its virulence in pigs. *Vet. Microbiol.* 174 391–398. 10.1016/j.vetmic.2014.09.027 25457365

[B39] Lincet H. Poulain L. Remy J. S. Deslandes E. Duigou F. Gauduchon P. (2000). The p21(cip1/waf1) cyclin-dependent kinase inhibitor enhances the cytotoxic effect of cisplatin in human ovarian carcinoma cells. *Cancer Lett.* 161 17–26. 10.1016/s0304-3835(00)00586-3 11078909

[B40] Miller C. N. Smith E. P. Cundiff J. A. Knodler L. A. Blackburn J. B. Lupashin V. (2017). A Brucella type IV effector targets the COG tethering complex to remodel host secretory traffic and promote intracellular replication. *Cell Host Microbe* 22 317–329. 28844886 10.1016/j.chom.2017.07.017 PMC5599354

[B41] Moreno E. (2014). Retrospective and prospective perspectives on zoonotic brucellosis. *Front. Microbiol.* 5:213. 10.3389/fmicb.2014.00213 24860561 PMC4026726

[B42] Myeni S. Child R. Ng T. Kupko J. Wehrly T. Porcella S. (2013). Brucella modulates secretory trafficking via multiple type IV secretion effector proteins. *PLoS Pathog.* 9:e1003556. 10.1371/journal.ppat.1003556 23950720 PMC3738490

[B43] Niu H. Kozjak-Pavlovic V. Rudel T. Rikihisa Y. (2010). Anaplasma phagocytophilum Ats-1 is imported into host cell mitochondria and interferes with apoptosis induction. *PLoS Pathog.* 6:e1000774. 10.1371/journal.ppat.1000774 20174550 PMC2824752

[B44] O’Callaghan D. Cazevieille C. Allardet-Servent A. Boschiroli M. L. Bourg G. Foulongne V. (1999). A homologue of the *Agrobacterium tumefaciens* VirB and *Bordetella pertussis* Ptl type IV secretion systems is essential for intracellular survival of *Brucella suis*. *Mol. Microbiol.* 33 1210–1220. 10.1046/j.1365-2958.1999.01569.x 10510235

[B45] Pan X. Luhrmann A. Satoh A. Laskowskiarce M. A. Roy C. R. (2008). Ankyrin repeat proteins comprise a diverse family of bacterial Type IV effectors. *Science* 320 1651–1654. 10.1126/science.1158160 18566289 PMC2514061

[B46] Postel E. H. Zou X. Notterman D. A. La Perle K. (2009). Double knockout Nme1/Nme2 mouse model suggests a critical role for NDP kinases in erythroid development. *Mol. Cell. Biochem.* 329 45–50. 10.1007/s11010-009-0110-9 19381783

[B47] Qin Q.-M. Pei J. Ancona V. Shaw B. D. Ficht T. A. de Figueiredo P. (2008). RNAi screen of endoplasmic reticulum–associated host factors reveals a role for IRE1α in supporting Brucella replication. *PLoS Pathog.* 4:e1000110. 10.1371/journal.ppat.1000110 18654626 PMC2453327

[B48] Robey R. B. Hay N. (2009). Is Akt the “Warburg kinase”?—Akt-energy metabolism interactions and oncogenesis. *Semin. Cancer Biol.* 19 25–31. 10.1016/j.semcancer.2008.11.010 19130886 PMC2814453

[B49] Sidhu-Muñoz R. Tejedor C. Vizcaíno N. (2020). Brucella ovisthe three flagellar loci of PA are dispensable for virulence in cellular models and mice. *Front. Vet. Sci.* 7:441 10.3389/fvets.2020.00441 PMC7410920 32851024

[B50] Sieira R. Comerci D. J. Sánchez D. O. Ugalde R. A. (2000). A homologue of an operon required for DNA transfer in Agrobacterium is required in Brucella abortusfor virulence and intracellular multiplication. *J. Bacteriol.* 182 4849–4855. 10.1128/jb.182.17.4849-4855.2000 10940027 PMC111363

[B51] Smith J. A. Khan M. Magnani D. D. Harms J. S. Durward M. Radhakrishnan G. K. (2013). Brucella induces an unfolded protein response via TcpB that supports intracellular replication in macrophages. *PLoS Pathog.* 9:e1003785. 10.1371/journal.ppat.1003785 24339776 PMC3855547

[B52] Starr T. Child R. Wehrly T. D. Hansen B. Hwang S. López-Otin C. (2012). Selective subversion of autophagy complexes facilitates completion of the Brucella intracellular cycle. *Cell Host Microbe* 11 33–45. 10.1016/j.chom.2011.12.002 22264511 PMC3266535

[B53] Starr T. Ng T. W. Wehrly T. D. Knodler L. A. Celli J. (2008). Brucella intracellular replication requires trafficking through the late endosomal/lysosomal compartment. *Traffic* 9 678–694. 10.1111/j.1600-0854.2008.00718.x 18266913

[B54] Taguchi Y. Imaoka K. Kataoka M. Uda A. Nakatsu D. Horii-Okazaki S. (2015). Yip1A, a novel host factor for the activation of the IRE1 pathway of the unfolded protein response during Brucella infection. *PLoS Pathog.* 11:e1004747. 10.1371/journal.ppat.1004747 25742138 PMC4350842

[B55] Thakur R. Yadav V. Kumar P. Chowdhury S. (2011). Mechanisms of non-metastatic 2 (NME2)-mediated control of metastasis across tumor types. *Naunyn-Schmiedeberg’s Arch. Pharmacol.* 384 397–406. 10.1007/s00210-011-0631-0 21556888

[B56] Wallimann T. (1994). Bioenergetics. Dissecting the role of creatine kinase. *Curr. Biol.* 4 42–46. 10.1016/s0960-9822(00)00008-7 7922310

[B57] Young B. M. Young G. M. (2002). YplA is exported by the Ysc, Ysa, and flagellar Type III secretion systems of *Yersinia enterocolitica*. *J. Bacteriol.* 184 1324–1334. 10.1128/jb.184.5.1324-1334.2002 11844761 PMC134849

[B58] Zheng R. Xie S. Lu X. Sun L. Zhou Y. Zhang Y. (2018). A systematic review and meta-analysis of epidemiology and clinical manifestations of human brucellosis in China. *BioMed Res. Int.* 2018 1–10. 10.1155/2018/5712920 29850535 PMC5937618

[B59] Zhou K. Wu B. Pan H. Paudyal N. Jiang J. Zhang L. (2020). One health approach to address zoonotic brucellosis: a spatiotemporal associations study between animals and humans. *Front. Vet. Sci.* 7:521 10.3389/fvets.2020.00521 PMC7492289 32984409

