# Supplementary figures and images for: Brucella abortus BspJ Is a Nucleomodulin That Inhibits Macrophage Apoptosis and Promotes Intracellular Survival of Brucella
Source: Front Microbiol. 2020 Nov 12;11:599205. doi: 10.3389/fmicb.2020.599205 (PMC7688787; doi:10.3389/fmicb.2020.599205)

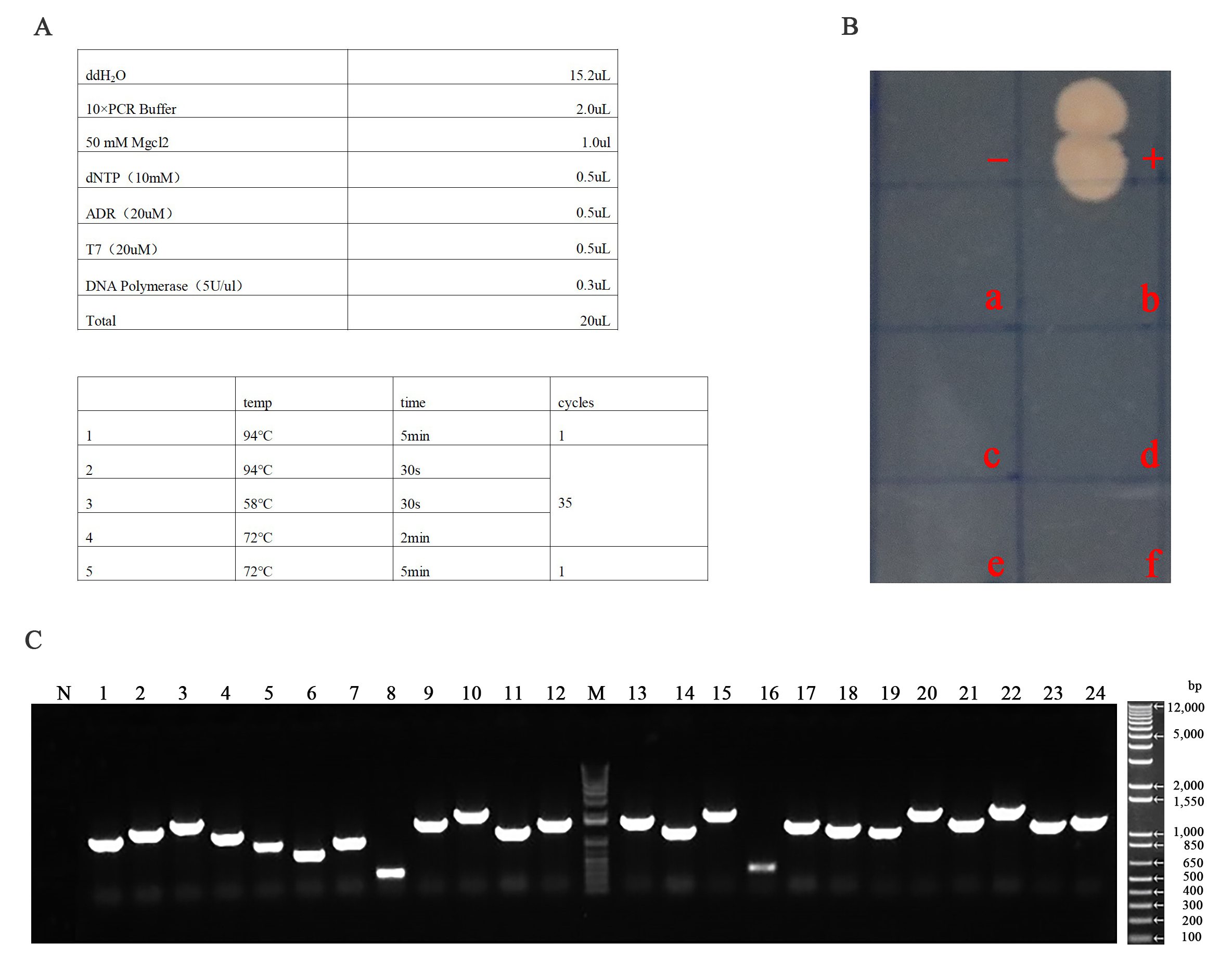

Supplement: Supplementary Figure 1 — (A) The detailed PCR parameters of identify the recombination rate and insert length of HEK293T cDNA library. (B) BspJ gene self-activation and toxicity detection. -. pGBKT7/pGADT7 negative control. +. pGBKT7-53/pGADT7-T positive control. (a-f) BspJ/pGADT7 test group. Growth on SD/–Ade/–His/–Leu/–Trp (QDO) medium. (C) Identifying the insert length in HEK293T cDNA library. N, Blank control. M, DNA marker. 1-24, PCR product of the sample. The results in the figure were obtained from three independent replicate experiments. Images are representative from three independent experiments. [file Image_1.JPEG]

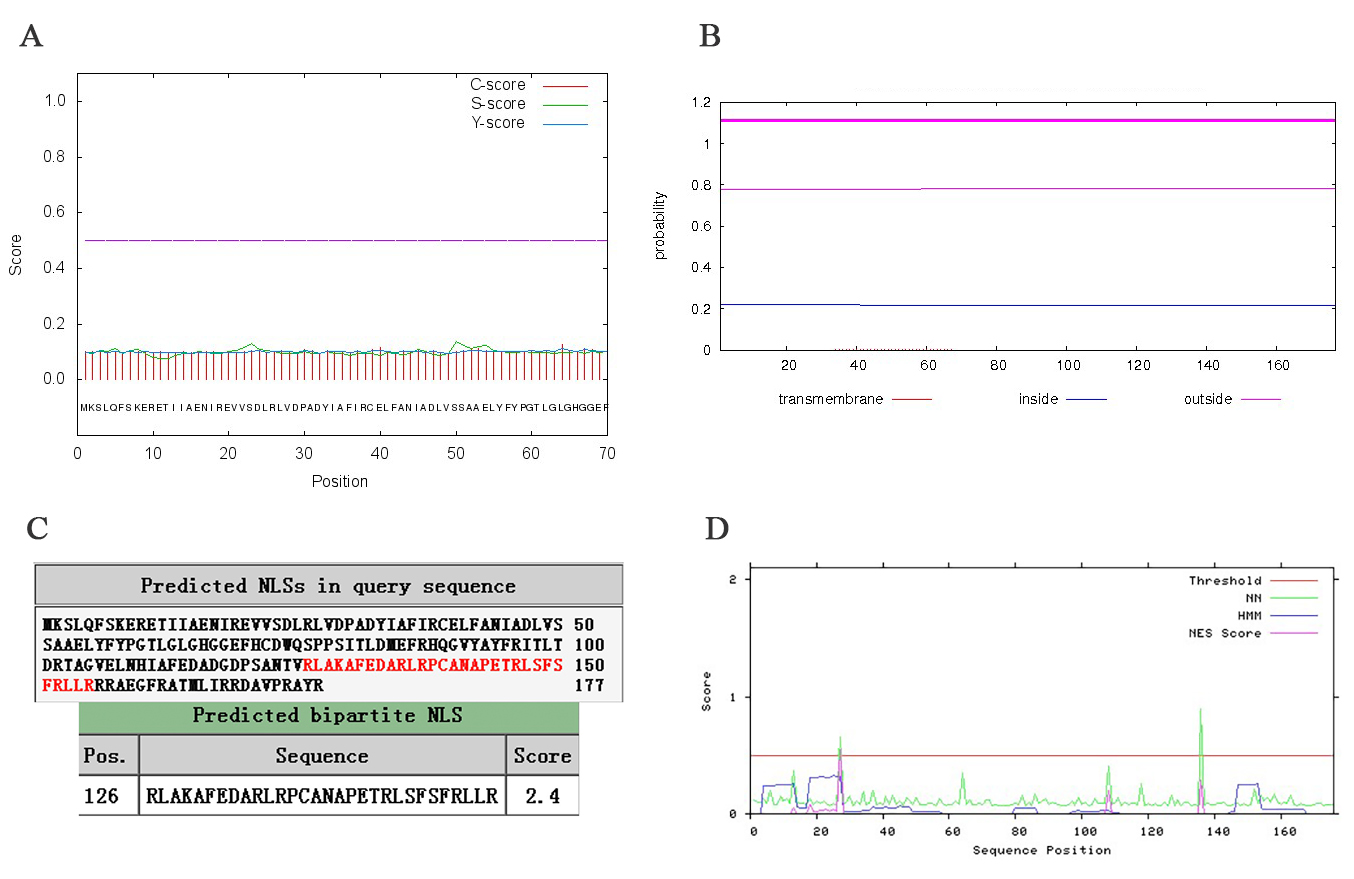

Supplement: Supplementary Figure 2 — (A) The result of BspJ signal peptide prediction. (B) The result of BspJ transmembrane structure prediction. (C) The result of BspJ protein nuclear localization signal (NLS) prediction. (D) The result of BspJ protein nuclear export signal (NES) prediction. [file Image_2.JPEG]

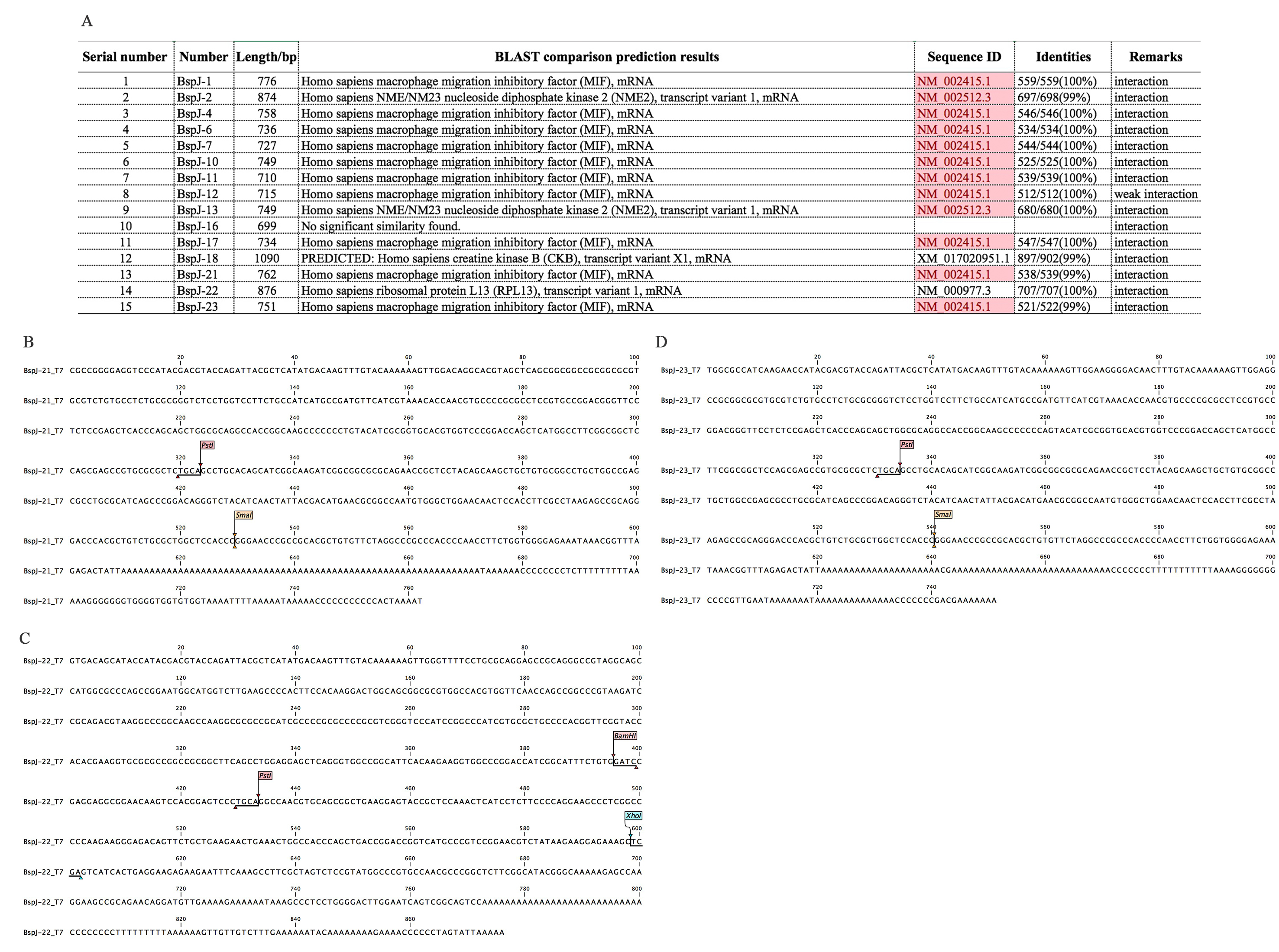

Supplement: Supplementary Figure 3 — Sequencing data and statistical results of 15 potentially interacting proteins with BspJ. [file Image_3.JPEG]

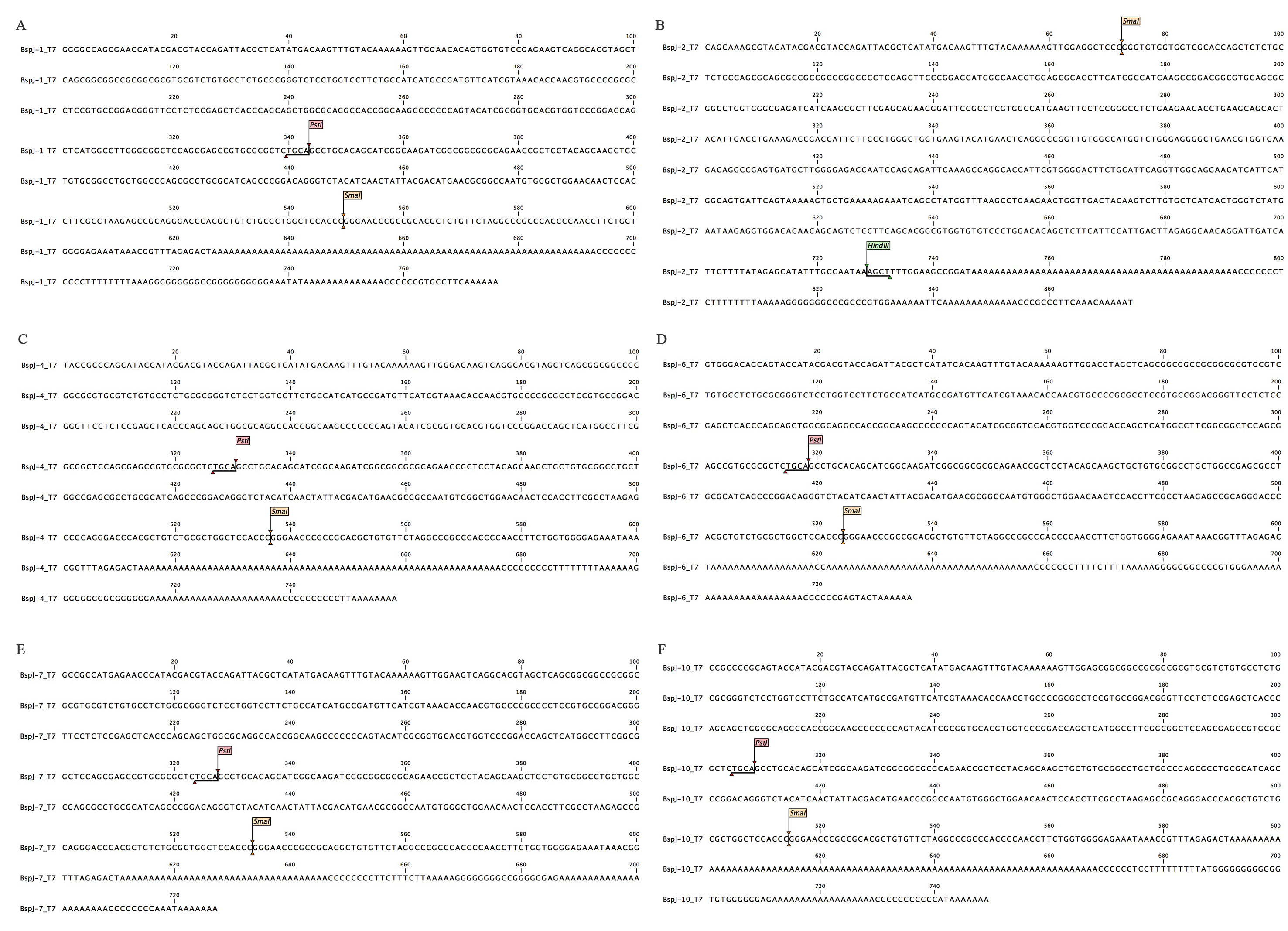

Supplement: Supplementary Figure 4 — Sequencing data and statistical results of 15 potentially interacting proteins with BspJ. [file Image_4.JPEG]

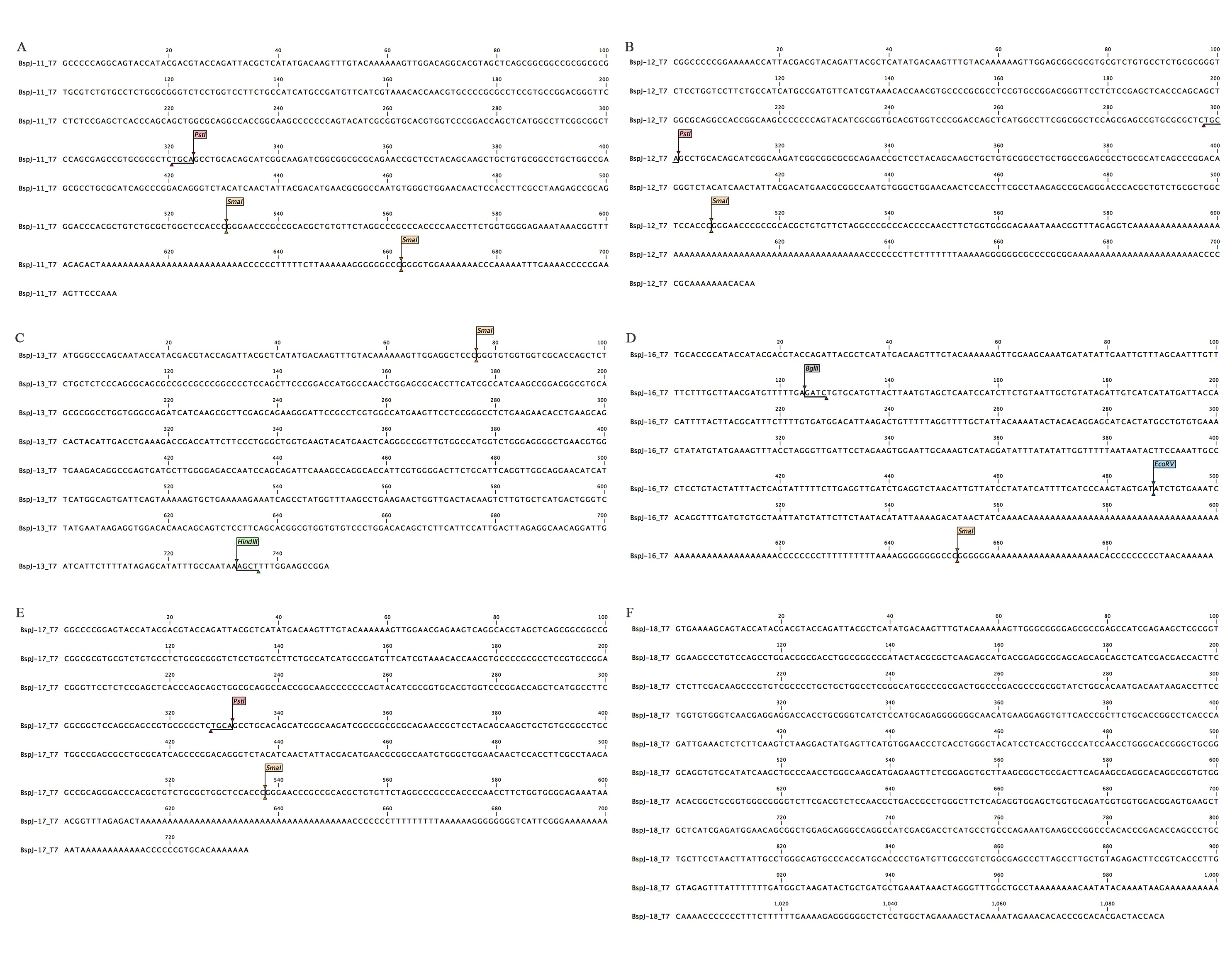

Supplement: Supplementary Figure 5 — Sequencing data and statistical results of 15 potentially interacting proteins with BspJ. [file Image_5.JPEG]

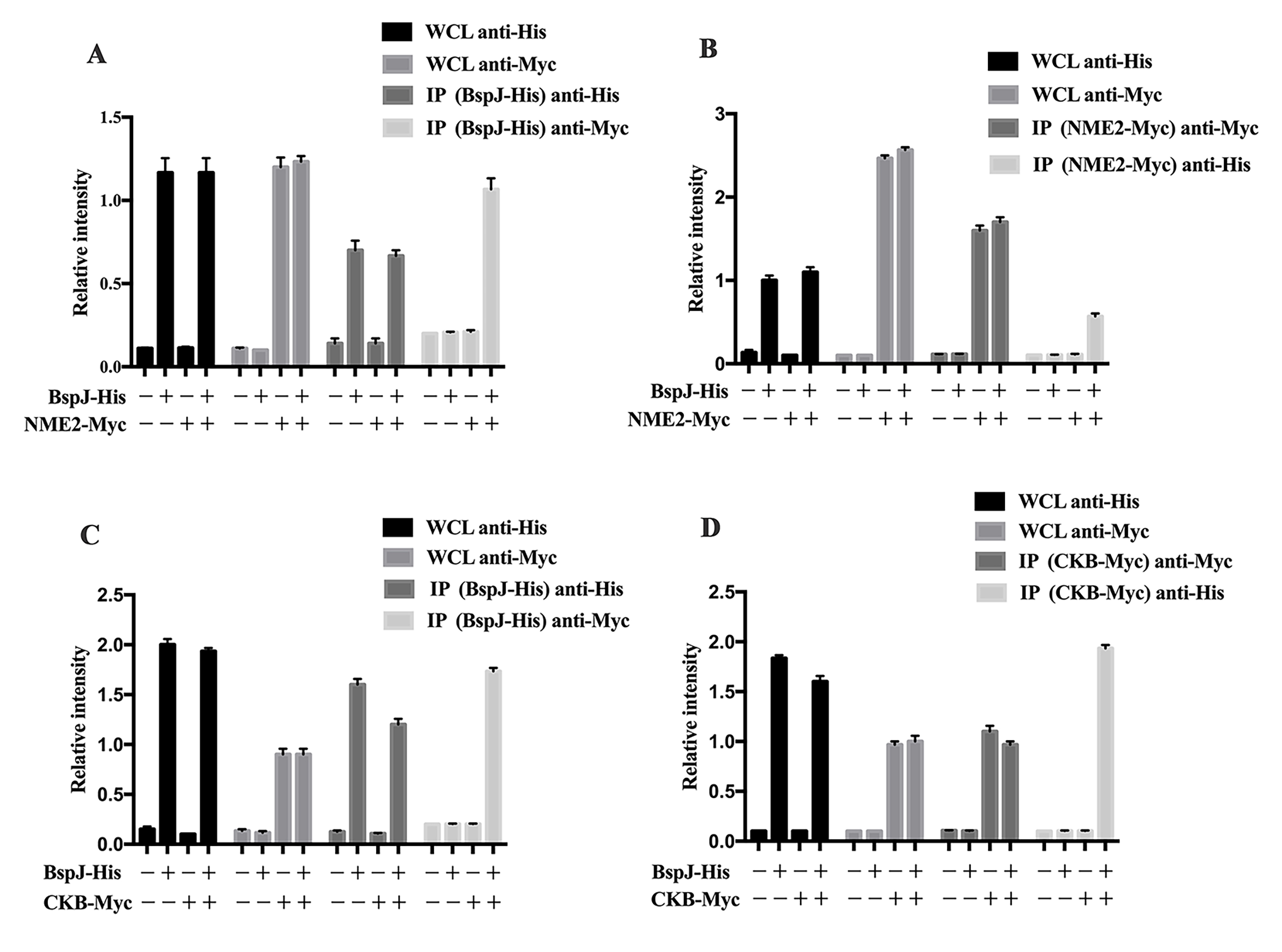

Supplement: Supplementary Figure 6 — Relative intensity for BspJ-His and NME2-Myc, BspJ-His and CKB-Myc were semi-quantified using ImageJ software. Images are representative from three independent experiments. [file Image_6.JPEG]

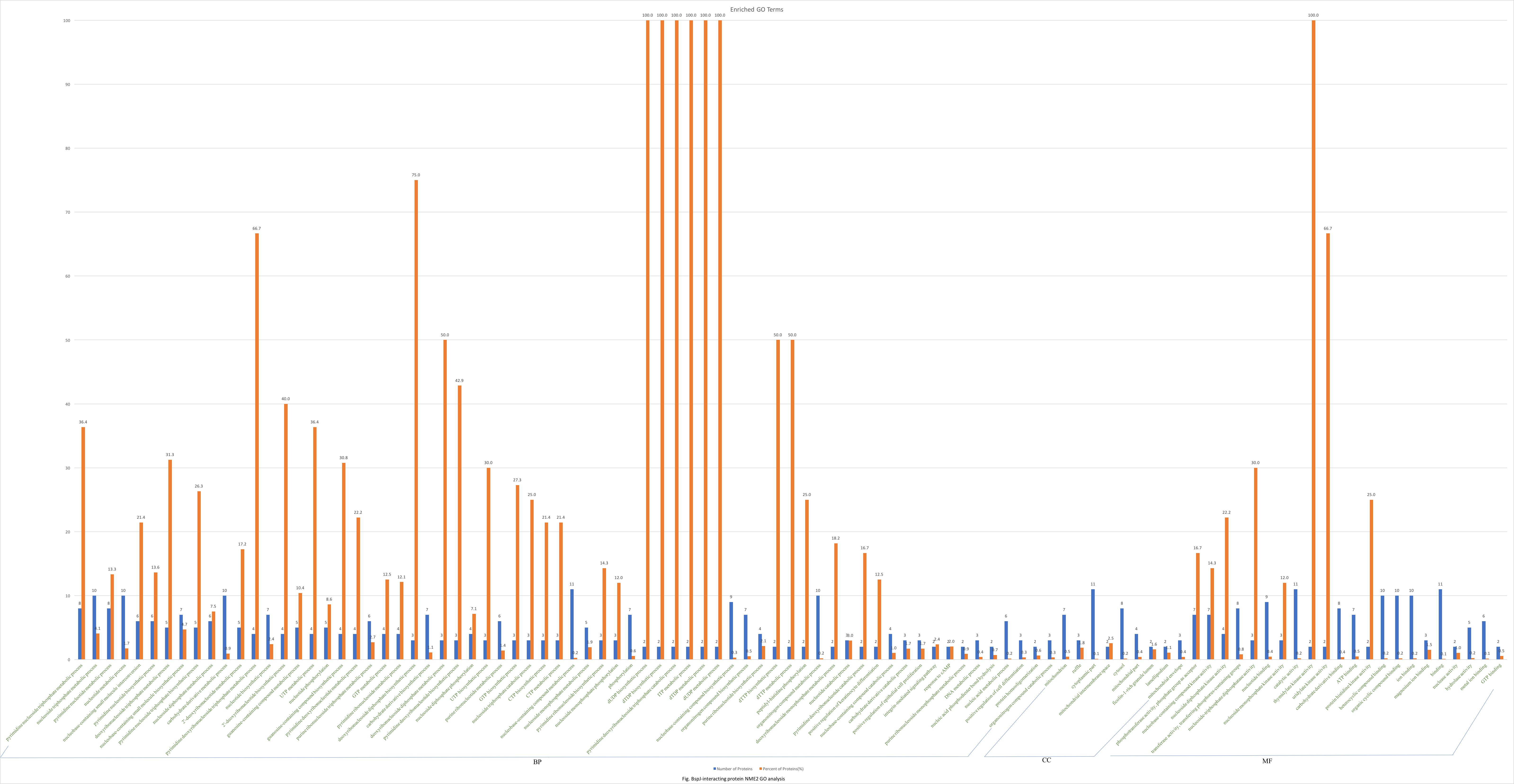

Supplement: Supplementary Figure 7 — The details GO analysis items of NME2. [file Image_7.TIF]
